# Comprehensive Systematic Review of Poly-L-Lactic Acid in Facial Clinical Application

**DOI:** 10.1007/s00266-025-05030-4

**Published:** 2025-06-27

**Authors:** Luiz Eduardo Toledo Avelar, Xiaoqing Yan, Daniel Bråsäter

**Affiliations:** 1 Private Clinic, Belo Horizonte, Brazil; 2 Beijing Tsinghua Changgung Hospital, Beijing, China; 3 Galderma, Uppsala, Sweden

*Level of Evidence I* This journal requires that authors assign a level of evidence to each article. For a full description of these Evidence-Based Medicine ratings, please refer to the Table of Contents or the online Instructions to Authors www.springer.com/00266.

With great interest, we have read the article published by Xu et al., titled “Comprehensive Systematic Review of Poly-L-lactic Acid in Facial Clinical Application” [1]. A literature review with the study objective to conduct a systematic review on the therapeutic efficacy and safety of PLLA in clinical applications for facial treatments per pre-defined inclusion and exclusion criteria resulting in a final analysis of only 20 articles is proposed.

First, it’s worth highlighting that lactic acid exists in different stereoisomers of polylactic acids known as poly-L-lactic acid (PLLA, Sculptra), poly-D-lactic acids, poly-D,L-lactic acids (PDLLA, Aesthefill) and polylactic acids (PLA, Löviselle), applied as various aesthetic injectables globally per regional/local authority approvals [2, 3]. All available polylactic acids in aesthetics differ in particle size, shape, physicochemical and stereochemistry characteristics [4] as well as differ in product compositions, all impacting clinical outcomes [5] predominantly efficacy and safety.

Beside the caveat with the review generalizing “PLLA” including PDLLA as well PLA despite significant differences, the review also fails describing the different “PLLA” injectable compositions leading to misleading descriptions of their vial contents; while it is correct that a Sculptra vial contains 150 mg of PLLA-SCA, a dry powder Sculptra vial also includes the excipients mannitol (127.5 mg) and sodium carboxymethylcellulose (90 mg) provided in an air free vial with a total dry powder weight of 367.5mg. Löviselle on the other hand is described by the authors as a 340 mg/vial in the same context describing Sculptra as a 150mg/vial which is misleading information, and we compare the Sculptra-PLLA vial content with the full dry weight of Löviselle (340 mg/vial), in which per instructions for use (Löviselle) contain 150 mg PLA, 145 mg mannitol and 45 mg CMC.

The authors also disregard the duration/longevity of effect with the selected literature (Löviselle study durations: 6-month since last injection/unknown vs. Sculptra study durations: up to 36 months) which is a key feature and clinical outcome of PLLA injectables. Despite the mentioned fundamental differences, the authors describe “*important clinical outcomes and safety on the clinical facial application of PLLA*” implying that PLLAs are the same which is misleading and based on a biased, unbalanced and an incomplete literature review, leading to shortcomings in the existing published work.

Worth highlighting is the imbalance between the literature review and the author discussion and conclusions, leading to a biased (not assessed despite using MINORS scale nor reported in the article) and misleading conclusion for “PLLAs” in general. Of 20 articles included per selection criteria in the literature review published up to 31 July 2023:

- 75% of the literature review refer to PLLA-SCA (Sculptra) clinical outcomes (efficacy (N: 425), safety (N: 611), and treatment satisfaction and psychological assessment (N:456)) capturing 15 out of 20 publications, and approximately 632 patients included in either Randomized Clinical Trials (RCT) or Non-Randomized Clinical Trials (NRCT).
- 100% of the literature referring to PLLA reconstitutions are PLLA-SCA (Sculptra) references.
- 20% refer to PLLA (Löviselle) from four (4) NRCT out of 20 publications and capture approximately 193 patients with limited data for efficacy (N: 73 distributed in three separate NRCTs), safety (N: 168), and treatment satisfaction and psychological assessment (N: 193)
- 5% refer to PDLLA (Aesthefill) from one (1) NRCT out of 20 publications capturing 30 patients and should be ignored when reviewing PLLAs comprehensively in the literature and per study objective/title of the article due to fundamental physiochemical differences [4].

Of concern is also that the authors in their literature review have excluded eligible and relevant publications, i.e., Palm et al. [6] per scope of search. Excluding data describe significant safety and reconstitution data accounting for patient outcomes in approximately 1000 patients related to PLLA-SCA (Sculptra), leaving doubt on the methodology, search terms, and objectivity of the review leading to an incomplete conclusion in the “comprehensive” literature review by the authors.

PLLA reconstitution processes are beside clinical outcomes key practical and usability parameters for injectors. As the reconstitution process is part of the PLLA injectable development, it requires significant science and research to prove that physiochemical parameters aren’t impacted [7] by the reconstitution process, as well describe effectiveness and safety through studies [6] (Sculptra, [8, 9] described and included in Xu et al. [1]). The article is misleading when describing “PLLA” reconstitutions, as all references in the article apply to PLLA-SCA (Sculptra) and none support immediate use for Löviselle confusing the interpretation of the article in the discussion and conclusion and misleading the reader. Most appropriate would be to refer to each injectable’s instruction for use (IFU) and their individual reconstitution processes, based on science and data for compliant use and with the focus on patients’ safety.

Lastly, the fact is that English has become the *lingua franca* of the international scientific community and reflecting on the article methodology of the literature review to be published in an international journal; all references referring to Löviselle are in Chinese. This becomes a major hurdle and counterproductive for a non-native Chinese scientific community for an objective review of the systemic literature review as based on the literature. A major shortcoming is the limited data listings and summaries in the article to be cross-referenced for an international scientific community reading an English international scientific journal and an international peer-reviewed publication.

Taken together, the systematic literature review on four (4) randomized clinical trials (RCT) and 16 NRCTs based predominantly (75%) on PLLA-SCA (Sculptra) data for efficacy, safety and satisfaction as well as PLLA-SCA (Sculptra) being the sole (100%) reference for immediate reconstitution shows a biased and unbalanced application of PLLA-SCA (Sculptra) specific data to all “PLLA” injectables in general. Clearly per the review, the only “PLLA” with efficacy and safety beyond 6 months after the last injection is PLLA-SCA (Sculptra), describing clinical outcomes beyond 2 years. PLLA-SCA is also the only PLLA with randomized clinical trial data per the review and the only PLLA with clinical evidence for immediate use. The literature review contains several misleading comparisons addressed in this letter, ignoring fundamental differences in physicochemical and stereochemistry characteristics (PLLA: Sculptra, PDLLA: Aesthefill, PLA: Löviselle), leading to an inappropriate generalization of polylactic acid injectables, which may mislead both aesthetic injectors and patients around clinical outcomes, treatment expectations and tolerability (adverse events), risking patients’ safety.
